# PeFoMed: Parameter efficient fine-tuning of multimodal large language models for medical CXR

**DOI:** 10.1038/s41598-026-47871-2

**Published:** 2026-04-21

**Authors:** Gang Liu, Xiaotian Tang, Jinlong He, Pengfei Li, Zhaolin Chen, Shenjun Zhong

**Affiliations:** 1 College of Computer Science and Technology, Harbin Engineering University, Harbin, 150001 China; 2 Nansha Islands Coral Reef Ecosystem National Observation and Research Station, Guangzhou, 510300 China; 3 Monash Biomedical Imaging, Monash University, Melbourne, 3800 Australia

**Keywords:** Multimodal large language model, Medical visual question answering, Medical report generation, Generative model, Parameter efficient fine-tuning, Computational biology and bioinformatics, Health care, Mathematics and computing, Medical research

## Abstract

Multimodal large language models (MLLMs) represent an evolutionary expansion in the capabilities of traditional large language models, enabling them to tackle challenges that surpass the scope of purely text-based applications. Recent works investigate the adaptation of MLLMs as a universal solution to address medical multi-modal problems as a generative task. In this paper, we propose a parameter efficient framework for fine-tuning MLLMs, specifically validated on medical visual question answering (Med-VQA) and medical report generation (MRG) tasks, using public benchmark datasets. We also introduce an evaluation metric using the 5-point Likert scale and its weighted average value to measure the quality of the generated reports for MRG tasks, where the scale ratings are labelled by both humans manually and the GPT-4 model. We further assess the consistency of performance metrics across traditional measures, GPT-4, and human ratings for both VQA and MRG tasks. The results indicate that GPT-4-based semantic evaluation can provide a scalable supplementary signal for assessing generated outputs, yet they reveal a discrepancy when compared to conventional lexical similarity measurements. This questions the reliability of lexical similarity metrics for evaluating the performance of generative models in Med-VQA and report generation tasks.

## Introduction

Medical multimodal tasks involve the integration of both computer vision (CV) and natural language processing (NLP) techniques to analyze data from multiple modalities (i.e. image and text) to answer clinical-related questions as medical visual question answering (Med-VQA) tasks or generate textual reports from radiological images. Various deep learning models primarily approach medical multimodal tasks using dedicated models for each task^1^, for example using classification models for VQA^2–7^ that categorize the image-text representation into a predefined set of answers, and auto-regressive models for generating image reports^8–10^.

Recently, an alternative solution is treating medical multimodal tasks as generative tasks by using language models as decoders^11^, which provides a universal framework to tackle medical multimodal problems. There has been emerging research that uses Large Language Models (LLMs)^12–15^ for generating free-form text as answers on Med-VQA tasks,^16^ using proper prompting techniques. This type of models, namely Multimodal Large Language Models (MLLMs) have been actively studied and the early attempts, such as Med-Flamingo^17^ and LLaVA-Med^18^ have shown the performance on various medical multimodal tasks.

However, directly training MLLMs from scratch for solving medical multimodal tasks requires numerous computational resources and large-scale annotated data. In response to these challenges, we propose a parameter-efficient adaptation framework that uses Parameter-Efficient Fine-tuning (PEFT) techniques on MLLM foundation models for Med-VQA and medical report generation (MRG) tasks, namely the PeFoMed model. We utilize the pre-trained weights of a general domain LLM and ViT^19^, which have been adeptly trained on diverse datasets, and fine-tune them with medical image-caption pairs and downstream Med-VQA and medical reports datasets. In training, the vision encoder and LLM are frozen, and only the vision projection layer and the low-rank adaptation layer (LoRA)^20^ are updated, which results in a minimal footprint of trainable parameters. Specific prompting templates are designed for the above fine-tuning process.

Furthermore, in generative tasks like VQA and report generation, in addition to using conventional metrics, we leverage the GPT-4 model to evaluate the semantic similarity of generated answers or reports and compare it with human evaluations to examine consistency. For report generation, where the text can be lengthy, we employ a 5-point Likert scale to gauge the overall quality and coherence. This approach investigates the potential of GPT-4 as an accurate evaluation tool for large datasets, with carefully designed prompting templates.

The contributions of this work can be summarized as follows:

- We present a parameter-efficient method for fine-tuning general-domain foundation models for medical CXR applications, providing a generative adaptation pipeline for two representative downstream tasks such as Med-VQA and MRG.
- We provide a systematic evaluation analysis showing that conventional lexical similarity metrics may be insufficient for assessing generative medical vision-language models. GPT-4-based semantic evaluation offers a useful supplementary signal, but also exhibits task-dependent discrepancies with human judgments, especially for report generation.
- Extensive experiments on benchmark datasets demonstrate that PeFoMed achieves competitive performance across Med-VQA and MRG tasks with substantially reduced trainable parameters, highlighting its practical value under constrained computational resources.

The remainder of this paper is organized as follows. Section 2 reviews related studies on medical multimodal large language models, medical visual question answering, and medical report generation. Section 3 presents the proposed method, including the model architecture, fine-tuning strategy, and evaluation criteria. Section 4 describes the datasets, implementation details, and experimental results. Section 5 concludes the paper, and Section 6 discusses the limitations of this study.

## Related works

In this section, we briefly review recent studies most relevant to our work from three perspectives: medical multimodal large language models, medical visual question answering, and medical report generation.

### Medical multimodal large language model

Large language foundation models, such as GPT-3^21^, PaLM^22^, and LLaMA^13^ have demonstrated superior performance across a diverse range of medical-related NLP tasks. Previous works like ChatDoctor^23^, Doctorglm^24^ and Huatuo^25^, have yielded promising results in various medical NLP tasks. One step ahead, the latest works have leveraged both vision and language foundation models to resolve multimodal tasks in the medical domain aligns with this paradigm. Early attempts to address multimodal (i.e. vision and language) problems, like Visual Med-Alpaca^26^, that converts images to intermediate text prompts, combine with the question texts and feed them into the LLM for predicting answers. This type of method may be constrained by the pre-trained image caption model and fail to capture detailed information from images.

Alternatively, recent work integrates vision embeddings into language models as visual prompts to enhance text generation. LLM-CXR^27^ applied a pre-trained VQ-GAN^28^ to tokenize images and generate visual and language tokens in an autoregressive manner, and fine-tuned the entire LLM with these tokens. Another intuitive solution is to explicitly model the projection of image embedding space to LLM space that can produce LLM-aligned image embeddings^29^. Similarly, LLaVA-Med^18^ fine-tuned the projection layer and the entire LM on the GPT-4 generated instruction-tuning and downstream biomedical datasets. HealthGPT^30^ employs heterogeneous low-rank adaptation and a three-stage training strategy to adapt diverse knowledge into pre-trained LLMs.

Fine-tuning the entire LLM may not always be a practical solution when computing resources are constrained, instead, parameter-efficient fine-tuning techniques offer a balanced and efficient approach to adapt large pre-trained models to specific tasks while preserving the vast knowledge these models have already acquired. There have been some works that use PEFT techniques in various multimodality use cases^31,32^. Therefore, in our approach, we employ parameter-efficient fine-tuning methods on Med-VQA and MRG tasks, which minimizes the training costs while yielding robust results.

### Medical visual question answering

Med-VQA tasks can be categorized into classification tasks^2–6,33^ and generative tasks^17,18,29^. Classification-based Med-VQA predefines an answer candidate set. Although this approach can yield high performance on specific datasets, it concurrently limits the model’s capability to address open-ended questions. When integrated with LLMs, Med-VQA tasks transition to generative tasks. Li et al. explored the zero-shot setting of the GPT-4v model^34^ on Med-VQA dataset, where GPT-4v is a universal model and is not tailored to specific Med-VQA data types and tasks. Its performance on both open-ended and closed-ended question types is not comparable to the existing non-generative methods. Concurrently, Med-Flamingo^17^ extended from the base Flamingo framework^35^, forming an in-context learning strategy with interleaved medical image-text pairs to achieve a few shot capacity for Med-VQA tasks. LLaVA-Med^18^ represents the first effort to adapt multimodal instruction tuning for the biomedical domain, implementing end-to-end training to develop biomedical multimodal dialogue assistants, thereby achieving promising outcomes in medical image-text dialogue. CMID^36^ is a training-free inference-time intervention for Med-VQA that addresses attention shift and attention dispersion in LVLMs via contrastive mutual information decoding. MedAtlas^37^ is a benchmark framework for evaluating LLMs on realistic medical reasoning tasks, featuring multi-round VQA, multi-modal image joint reasoning, and high clinical fidelity across diverse imaging modalities.

### Medical report generation

Similar to image-captioning tasks, MRG generates text corresponding to medical images. As medical reports, the text generated by MRG is more professional than the conventional image-captioning task, and the text is relatively long and more complex. R2Gen^38^ produces radiology reports through a memory-driven transformer employing relational memory to capture key information throughout the generation process. Additionally, R2Gen employs conditional layer normalization to integrate memory within the transformer’s decoder. Chen et al.^10^ observed cross-modal data bias and applied cross-modal causal intervention to reduce this bias in medical reports and images, achieving notable results in medical report datasets. ITHN^9^ learns discriminative features between images and reports by separately learning images and their hard negatives, thereby capturing fine-grained details in multimodal data. Chen et al.^8^ addressed the discrepancy between medical images and text data by designing a distiller that leverages both prior and posterior knowledge to identify specific abnormalities in images and assimilate prior medical knowledge, yielding high performance in datasets such as IU-Xray. HiMed-RL^39^ is a hierarchical medical reward learning framework that addresses clinical hallucinations in report generation by deconstructing reward learning into token-level fluency, concept-level factual grounding, and semantic-level diagnostic consistency. FAST-MRG^40^ is a hybrid encoder-decoder architecture for medical report generation that combines a distillation-enriched transformer encoder with a generative pre-training transformer decoder to produce paragraph-level reports at low computational cost.

Furthermore, Yang et al. introduced MedXChat^41^, a model utilizing LLMs for chest X-ray multimodal tasks, which demonstrated effectiveness in generating chest X-ray medical reports. However, these studies often concentrate on a single MRG task or a specific type of medical image, like chest X-rays, employing general-domain text generation metrics for evaluation. Such metrics cannot accurately represent the quality of the generated medical reports.

## Methods

In this section, we will introduce the model architecture used in the work and the training strategy which involves two stages of fine-tuning. Subsequently, we will delineate the evaluation criteria adopted and the metrics proposed for MRG.

**Fig. 1:**
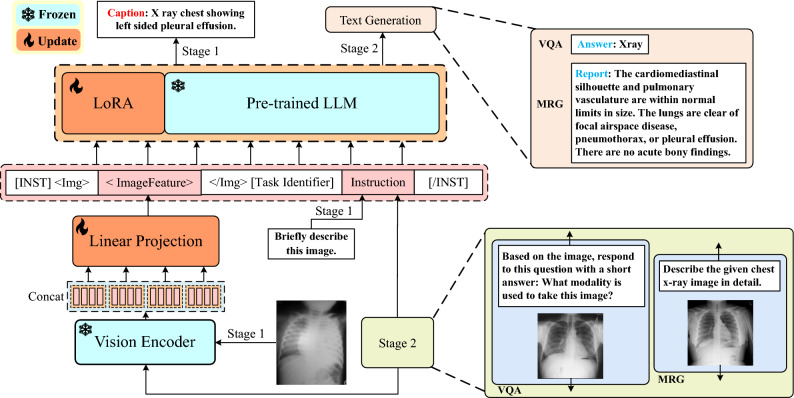
The architecture of the model.

### Model architecture

The model is composed of three parts: the vision encoder, a pre-trained LLM for processing multimodal inputs and generating answers, and a single linear layer for projecting embeddings from visual encoding space to LLM space, as shown in Fig. 1. A ViT type of visual backbone, EVA^19^ is used to encode image tokens into visual embeddings, where the model weights are frozen during the entire fine-tuning processes. We group four consecutive tokens into one single visual embedding to effectively reduce resource consumption, by concatenating on the embedding dimension. The grouped visual tokens are then fed into the projection layer and output embeddings (of length 4096) in the LLM space. Subsequently, multimodal interaction is implicitly achieved through self-attention over the shared multimodal token sequence in the decoder. A multimodal prompt template is designed to include both visual and question information and passed to the pre-trained LLM, LLaMA2-chat(7B)^15^ as the decoder for generating answers. We adopt the low-rank adaptation (LoRA) technique^20^ in the LLM for efficient fine-tuning, where the other parts of the LLM are entirely frozen during the downstream fine-tuning. A beam search with a width of 1.

The multimodal prompt incorporates input images, questions and dedicated tokens for downstream tasks. In Fig. 1, image features derived from linear projection are denoted as *<ImageFeature>*, and the corresponding questions are the text instructions. Special tokens, such as *[VQA]* for the Med-VQA task and *[report]* for the MRG task, serve as task identifiers. This forms the complete multimodal instructional template as:*[INST]<img><ImageFeature></img>[Task Identifier] Instruction [/INST]*.

### Model training

We use the weights from MiniGPT-v2^31^ that are pre-trained on datasets in general domains, and further fine-tune the models using multimodal medical datasets in two stages. We adopt an efficient fine-tuning technique, LoRA^20^, to only update a small part of the entire model. The details can be seen below:

*Stage 1: Fine-tuning with Image Captioning.* During this stage, we fine-tune the model using four medical image-caption datasets, ROCO^42^, CLEF2022^43^, MEDICAT^44^ and MIMIC-CXR^45^. These datasets consist of medical image-caption, the captions are text descriptions of the corresponding images and have a variety of lengths. We use the prompt template: *<Img><ImageHere></Img>[caption]<instruction>*, and the instruction prompt used is randomly selected from a pool of four candidates, the motivation is to reduce overfitting to a single fixed phrasing and improve robustness to instruction variations, e.g. “Briefly describe this image”. During training, only the linear projection layer and the LoRA layer in the LLM are fine-tuned, while the other parts of the models are frozen.

*Stage 2: Fine-tuning on VQA and Report Generation.* In the second stage, the model undergoes fine-tuning on the Med-VQA and MRG datasets. Specifically, for Med-VQA, the VQA-RAD^46^, SLAKE^47^, and PathVQA^48^ datasets are used, while the MIMIC-CXR^45^ dataset is utilized for the downstream MRG task. We adopt the following template, “*[INST]<img><ImageFeature></img>[Task Identifier] Instruction [/INST]*”, where in *[Task Identifier]* is substituted with *[VQA]* or *[Report]* according to the downstream tasks. For Med-VQA, the instruction prompt used in our experiment is: *Based on the image, respond to this question with a short answer: {question}*, where *{question}* is the question corresponding to the given medical image. The motivation for generating short answers is to validate against the annotated ground truth labels in VQA datasets where the answers are mostly short in both open-ended and closed-ended QA pairs. For MRG task, the instruction prompt is randomly selected from an instruction pool, for example: *Describe the given chest x-ray image in detail*. Similarly, at this stage, we also keep the vision encoder and the LLM frozen while only updating the linear projection and LoRA layer in LLM.

**Table 1:** Examples of model prediction on open-ended questions.

Question	Ground truth	Prediction
What kind of image is this?	x-ray	Chest x-ray
The mass is found in which part of the pancreas?	Pancreatic head	Head
Is the spleen present?	On patient’s left	Yes
Are pleural opacities located on the left, right, or both sides of the lung?	Both	Bilateral

### Evaluation metrics

In the experiment, we observed that the generated answers and the ground truth labels in the VQA task may not always match exactly on a word-by-word basis, particularly for open-ended questions. As shown in Table 1, one common pattern we observed is an inclusive relationship between the ground truth and the generated answer. For example, in the first case, the ground truth is “x-ray,” while the generated answer is “chest x-ray.” In conventional metrics, this would be falsely classified as an incorrect prediction. In the second example, the prediction, “head,” is semantically equivalent to the ground truth, “pancreatic head,” where organ information is presented in the question. Additionally, there are cases where the prediction and ground truth are synonyms, such as ‘both’ and ‘bilateral’ when asking questions about the sides of the lung.

To mitigate this issue, in our experiments, we used not only objective evaluation metrics but also the GPT-4 model to assess the semantic similarity of the generated answers or reports, comparing them with human annotations.

**Fig. 2:**
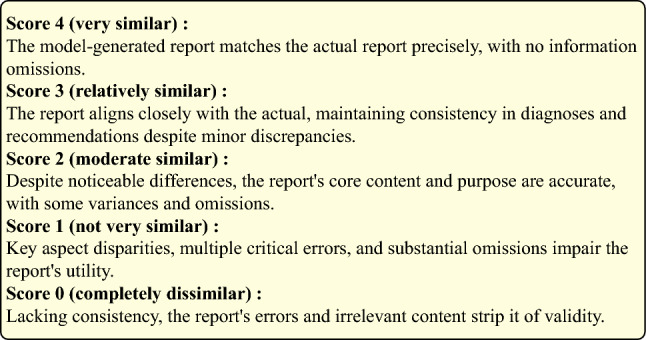
5-point Likert scale for semantic similarity, used to evaluate medical reports.

*The objective evaluation metrics* used include ACC, BLEU, METEOR, ROUGE-L, and Clinical Efficacy (CE) metrics, which assess the accuracy of generated reports in describing clinical abnormalities. The CE metrics consist of Precision, Recall, and F1 scores. These metrics require the use of the CheXpert toolkit to extract labels from predictive reports and ground truth, followed by a comparison of the presence status of important clinical observations to capture the diagnostic accuracy of the generated reports.

*Semantic Similarity Using GPT-4:* We explore the use of GPT-4 as the measurement engine to evaluate the semantic similarity between the ground truth text and its generated counterparts from the proposed models, which are then validated against human-labeled data. In text generation tasks, where the generated content is relatively lengthy, using a binary rating may not be ideal. Instead, a 5-point Likert scale for semantic similarity provides a more fine-grained validation of the results. As shown in Fig. 2, it consists of five levels: 0, 1, 2, 3, and 4, with each level representing specific evaluation criteria. The quality of the generated reports progressively improves from level 0 (completely dissimilar) to level 4 (very similar). We calculate the weighted average score of the similarity metric (WASM) as follows:

1 $$\begin{aligned} WASM = \frac{ \sum _ { i = 0 } ^ { 4 } ( i \cdot N _ { i } ) }{ \sum _ { i = 0 } ^ { 4 } N _ { i } }, \end{aligned}$$

where *i* denotes the ith level and $N _ {i}$ denotes the number of samples in the ith level. In this study, we use WASM as a descriptive summary statistic for ordinal Likert ratings. While WASM assumes interval-level data, it is important to clarify that it is employed solely as a statistical reference to approximate the central tendency of ratings across multiple raters, rather than as a clinical metric. This approach serves as a useful approximation when exploring general trends across raters.

Note that using GPT-4V as a generative system (image $\rightarrow$ answer) differs from using GPT-4 as a text-based semantic judge (GT + generated text $\rightarrow$ similarity). The evaluation task is more constrained and does not require extracting fine-grained clinical findings directly from images. For details on prompt-based safeguards and the use of LLMs as evaluators, please refer to Appendix C.

*Human evaluation protocol:* We prepared a spreadsheet containing all predictions with their associated ground truth. Ten raters (4 clinical staff and 6 medical students) independently evaluated all samples under a blinded and randomized protocol after guideline-based calibration.Inter-rater reliability was measured by Krippendorff’s $\alpha$ . For VQA, raters judged answer correctness (binary), with Krippendorff’s $\alpha$ = 0.807 (nominal), 95% CI [0.753, 0.842]. For MRG, we used a 5-point Likert scale (0–4) to assess semantic consistency between generated and ground-truth reports, with Krippendorff’s $\alpha$ = 0.698 (ordinal), 95% CI [0.629, 0.756] (Appendix A-Table 1).

### Experiments and results

In this section, we first describe the datasets and implementation details used in the experiments. We then present comparisons with existing methods on Med-VQA and MRG tasks, followed by ablation studies and additional analyses on evaluation consistency and transfer effects.

### Datasets

We utilized four datasets for stage 1 image-caption fine-tuning: ROCO^42^, CLEF2022^43^, MEDICAT^44^, and MIMIC-CXR^45^. ROCO, consisting of 87,952 radiological images and associated captions from PubMed Central, our study used only the training set. CLEF2022, offering broad coverage across medical fields, contains over 90,000 image-caption pairs. MEDICAT features over 210,000 image-caption pairs from PubMed Central. MIMIC-CXR features 377,110 radiology images and 227,835 reports from 64,588 patients. We used the official training set split.

Stage 2 fine-tuning for Med-VQA utilized three datasets: VQA-RAD^46^, SLAKE^47^, and PathVQA^48^. VQA-RAD comprises 315 radiologic images with 3,515 question-answer pairs across 11 question types such as abnormality and modality, featuring 104 axial CT scans of the abdomen, 104 head scans (CT/MRI), and 107 chest radiographs, split into 3,064 training and 451 test pairs. SLAKE, a bilingual Med-VQA dataset, provided us with the English subset containing 642 images and 7,032 pairs, divided as 4,918 for training, 1,053 for validation, and 1,061 for testing. PathVQA features 4,998 pathology images and 32,795 question-answer pairs. It includes eight question types: ’what’, ’where’, ’when’, ’how’, ’why’, ’whose’, ’which’, and ’how much’. The dataset was split into training, validation, and test sets in a 7:1:2 ratio.

The MIMIC-CXR dataset was utilized for stage 2 fine-tuning in MRG. MIMIC-CXR is a large publicly available dataset of chest radiographs in DICOM format with free-text radiology reports. The dataset contains 377,110 images corresponding to 227,835 radiographic studies performed at the Beth Israel Deaconess Medical Center in Boston, MA. The dataset was split into training, validation, and test sets according to the official proportions.

### Compute/training budget and parameter efficiency

All experiments were carried out using Python 3.9 on 4 NVIDIA Tesla A40 GPUs, each with 48GB of GPU memory. We initialized the model using the pre-trained weights of MiniGPT-v2. Throughout the training process, we only updated the linear projection layer and fine-tuned the LoRA layers (with a rank of 64) of the LLM.

In both fine-tuning stages, images are set to a resolution of $448\times 448$, and the maximum text length is set to 1024. We employed AdamW^49^ optimizer along with a cosine learning rate scheduler to train the model. For stage 1 fine-tuning, the learning rate was gradually decreased from an initial $1e^{-4}$ to $8e^{-5}$, the epoch is set to 3. In stage 2 fine-tuning, the learning rate is progressively lowered from $3e^{-5}$ to $1e^{-5}$, and the epoch is set to 50.

In total, PeFoMed updates 56.63M parameters, which is  0.81% of a 7B-scale backbone. Compared with full fine-tuning, updating only a small subset of parameters substantially reduces the optimization footprint, making the adaptation of MLLMs to medical imaging tasks more feasible under constrained compute resources.

### Comparison with the state-of-the-art methods

In this section, we present the results of our proposed models against the existing other models, on both Med-VQA and MRG tasks.

(a) * Medical visual question answering* For Med-VQA, as shown in Table 2, we compare the proposed model with other methods. Because prior methods use different backbones, optimization settings, and evaluation criteria, the comparisons in Table 2 should be interpreted as competitive reference results rather than strictly controlled one-to-one superiority claims. We briefly categorized existing models based on their decoder types, including non-LLM types such as classifiers, BERT^50^, and generative LLMs. For non-LLM approaches employing classifiers or BERT as decoders, the accuracy was computed through an exact match metric between the predicted answers and the ground truth text. On the other hand, the type of methods, like our method, used generative models that yield free-form text as answers using LLM as decoders, where the accuracy was measured differently. LLaVA-Med^18^ employed token-based recall for open-ended questions, while exact-match accuracy for closed-ended ones. In our work, we applied three accuracy metrics for the generated free-form answers: exact match accuracy as in the previous works, GPT-4 similarity evaluation and human manual evaluation in order to minimize the evaluation bias.

**Table 2:** The performance of various models on the VQA-RAD, SLAKE, and PathVQA datasets, where numbers in brackets represent standard deviations, indicating that the results come from the average of ten independent evaluations.

Methods	Type	Accuracy measurement	Trainable parameters	VQA-RAD			SLAKE			PathVQA		
				Open	Closed	Overall	Open	Closed	Overall	Open	Closed	Overall
MMQ^2^	Non-LLMs	Exact match	28.3M	52.0%	72.4%	64.3%	-	-	-	11.8%	82.1%	47.1%
MTL^4^			-	69.8%	79.8%	75.8%	80.2%	86.1%	82.5%	-	-	-
VQA-Adapter^3^			2.09M	66.1%	82.3%	75.8%	79.2%	83.7%	81.0%	-	-	-
M3AE^5^			-	67.2%	83.5%	77.0%	80.3%	87.8%	83.2%	-	-	-
M2I2^6^			262.15M	66.5%	83.5%	76.8%	74.7%	91.1%	81.2%	36.3%	88.0%	62.2%
MUMC^33^			211.06M	71.5%	84.2%	79.2%	81.5%	91.1%	84.9%	39.0%	90.4%	65.1%
ARL^51^			362M	67.6%	86.8%	79.2%	81.9%	91.4%	85.6%	-	-	-
LLaVA-Med(LLaVA)^18^	LLMs	Open: Token Recall Closed: Exact Match	7B	61.5%	84.2%	75.2%	83.1%	85.3%	84.0%	38.0%	91.2%	64.7% [t]
LLaVA-Med(Vicuna)^18^				64.4%	82.0%	75.0%	84.7%	83.2%	84.1%	38.9%	91.7%	65.3%
LLaVA-Med(Bio-CLIP)^18^				64.8%	83.1%	75.8%	87.1%	86.8%	87.0%	39.6%	91.1%	65.4% [b]
GPT-4v^34^		Exact Match	-	-	61.4%	-	-	-	-	-	-	-
PeFoMed (ours)		Exact Match	56.63M	62.6%	87.1%	77.4%	77.8%	88.7%	82.1%	35.7%	91.3%	63.6%
		Human Evaluation		77.7%(3.2%)	87.6%(0.2%)	83.7%(1.3%)	-	-	-	-	-	-
		GPT-4 Evaluation		79.9%(1.2%)	87.5%(0.0%)	84.4%(0.5%)	83.1%(0.3%)	88.7%(0.0%)	85.3%(0.2%)	45.7%	91.3%	68.6%

On the VQA-RAD dataset, our LLM-based method was close to the performance of the dedicated VQA models, with an overall accuracy of 77.4%, while outperforming the existing methods on closed-ended questions (87.1%), under the same exact-match accuracy metric. For open-ended questions, our MLLM-based model achieved 62.6%, compared to non-LLM dedicated visual language models, such as M3AE (67.2%)^5^ and MUMC (71.5%)^33^ In comparison, the early attempt to use LLM as answer decoders by the LLaVA-Med model series^18^ achieved overall accuracy of 75.8%, specifically, 84.2% for closed-ended questions and 64.8% for open-ended questions. It is important to note that LLaVA-Med measures accuracy for open-ended and closed-ended questions differently, i.e. measuring token recall rate for open-ended questions, and conventional classification accuracy for closed-ended types. Furthermore, a recent study validated the capability of GPT-4v (without fine-tuning) on the VQA dataset, and reported an accuracy of 61.4% on closed-ended questions, where our model achieved 87.1% in comparison.

As discussed in the method section, accuracy measured using the exact-match metric may not reflect the true performance in an MLLM-based generative setup. Therefore, we cross-validated the results using both GPT-4 and human evaluation methods, which were collected from 10 human annotators, and 10 independent inferences on the GPT-4 model with different seeding choices. Table 2 presents the results, indicating that the accuracies measured by human annotators (87.6%) and GPT-4 (87.5%) align closely with the exact match metric (87.1%). However, a significant discrepancy is observed in open-ended questions, where using GPT-4 evaluation yields an accuracy of 79.9%, closely mirroring the human annotations (77.7%) and surpassing the exact matches. This discrepancy raises concerns about the suitability of the exact match metric for evaluating open-ended questions and MRG tasks.

**Fig. 3:**
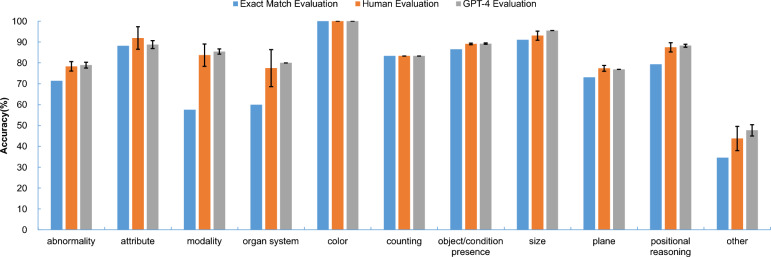
The accuracy of different question types on the VQA-RAD dataset by different evaluation methods.

We further analyzed the impacts of different evaluation methods across the pre-defined question types within the VQA-RAD dataset. Figure 3 shows the accuracy under the three distinct evaluation methods and the standard deviations for human and GPT-4 evaluations. The results suggest that accuracy measurements from human and GPT-4 evaluations are closely aligned across different question types, while GPT-4 has a consistently low variability compared to human annotators. A noticeable disparity can be seen between the results of human and GPT-4 evaluation methods and the exact match evaluation, for question types of ’abnormality’, ’modality’, and ’organ system’. Besides, regardless of the evaluation metrics, the model performed relatively well on question types, like ‘color’, ‘attribute’ and ‘size’. Questions associated with ‘abnormality’, ‘modality’ and ‘organ system’ seem to be challenging for our approach.

**Table 3:** The accuracy of different phrase types on the VQA-RAD dataset by different evaluation methods, para denotes the parameterized form phrase.

Evaluation methods	Phrase	
	Freeform	Para
Exact match evaluation	72.7%	87.4%
Human evaluation	81.5%(1.8%)	88.2%(0.4%)
GPT-4 evaluation	80.8%(0.6%)	92.2%(0.3%)

**Table 4:** The accuracy of different image organs under different evaluation methods on the VQA-RAD dataset.

Evaluation methods	Organ		
	CHEST	HEAD	ABDOMEN
Exact match evaluation	81.0%	79.0%	72.2%
Human evaluation	86.4%(1.0%)	85.4%(2.8%)	79.4%(1.9%)
GPT-4 evaluation	87.4%(0.0%)	86.8%(1.3%)	79.3%(0.7%)

A similar discrepancy can be seen on different phrase types (i.e. free-form and parameterized form) in the VQA-RAD dataset. The free-form question refers to a type of question or answer format that does not adhere to a predefined structure or set of possible responses, and are usually open-ended, allowing for a wide range of natural language expressions. Parameterized form phrase uses a structured representation of a question or answer that includes placeholders for specific parameters. For example, In the phrase, “What is the size of the [anatomical structure]?” where “[anatomical structure]” is a placeholder that can be replaced with different anatomical structures such as “heart”, “lung”, or “tumor” to generate specific questions like “What is the size of the heart?” As shown in Table 3, the discrepancy of exact-match and GPT-4 or human evaluation in free-form questions is much larger than the parameterized ones. As shown in Table 4, among the three organ types, the largest discrepancy between human and GPT-4 evaluations is merely 1.4%, with the smallest being 0.1%. The smallest gap with exact match evaluation reaches 5.4%.

**Fig. 4:**
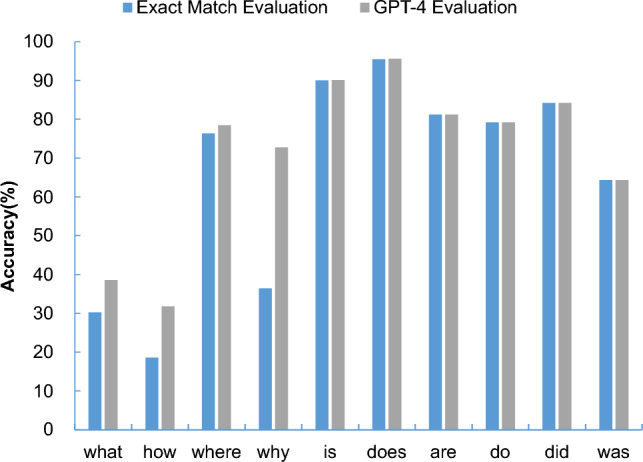
Accuracy of different evaluation methods for different types of questions on the PathVQA dataset.

Similarly, we observed significant performance gaps of accuracy measurements for open-ended questions on both the SLAKE and PathVQA datasets, using GPT-4 and exact-match evaluations. On the SLAKE dataset, our model achieved an overall accuracy of 82.1% (with exact-match evaluation) and 85.3% (using GPT-4 evaluation), showcasing a competitive performance across all evaluated models. On the PathVQA dataset, the largest among the three VQA datasets, featuring a test set of 6761 samples, there is an even larger discrepancy of 10% for open-ended questions between the two evaluations.

Using GPT-4 semantic similarity evaluation, the results demonstrate a strong consistency across multiple inferences on the VQA-RAD and SLAKE datasets. The largest standard deviation across 10 independent runs is only 1.2%, compared to the results of 10 independent human annotators (3.2%) for open-ended questions. On closed-ended questions, the standard deviations on GPT-4 evaluation over multiple runs between generated answers and ground truth is nearly 0 on both VQA-RAD and SLAKE datasets.

As illustrated in Fig. 4, we provide a detailed analysis of the various types of questions contained in the PathVQA dataset (A more detailed analysis of the Slake dataset can be found in Appendix B). For most of the closed-ended question types in this dataset, like the questions of ‘is’, ‘does’ and ‘did’, the results using GPT-4 evaluation have almost identical results with the exact-match evaluation. On the other hand, observations revealed that for more abstract question types, including ’what’, ’how’, and ’why’, the accuracy measurements using exact match evaluation significantly differ from the counterparts using GPT-4 semantic similarity evaluation. This indicates that exact match evaluation may not be sufficiently effective in assessing the performance of MLLMs on more complex open-ended question types.

Besides, it is worth highlighting that with the adoption of parameter-efficient fine-tuning, our model featured notably fewer trainable parameters (56.63M) in comparison to LLaVA-Med (7B parameters), which made our method a significantly more efficient framework for fine-tuning MLLMs.

**Table 5:** Performance of various models on the MIMIC-CXR.

Methods	Precision	Recall	F1	BLEU-1	METEOR	ROUGE
M2KT^52^	0.420	0.339	0.352	0.386	-	0.274
KiUT^53^	0.371	0.318	0.321	0.393	0.160	0.285
ME^54^	0.364	0.309	0.311	0.386	0.152	0.291
MAN^55^	0.411	0.398	0.389	0.396	0.151	0.274
PromptMRG^56^	0.501	0.509	0.476	0.398	0.157	0.268
PeFoMed (ours)	0.461	0.452	0.458	0.382	0.157	0.286

(b)* Medical report generation* As shown in Table 5, our MLLM-based model was evaluated on MRG tasks using the MIMIC-CXR dataset. We evaluated the performance with the commonly used metrics: BLEU, METEOR, ROUGE-L and CE metrics (Precision, Recall, F1). In terms of Clinical Efficacy, PeFoMed outperforms most medical report generation models, but falls short of existing state-of-the-art (SOTA) models.

**Table 6:** Semantic similarity for 100 randomly selected samples from the MIMIC-CXR dataset, assessed through both human evaluation and GPT-4 evaluation, respectively.

Evaluation methods	0	1	2	3	4	WASM$\uparrow$
Human evaluation	7.75(5.55)	23.38(12.81)	21.88(5.64)	29.13(14.73)	17.25(11.07)	2.24(0.42)
GPT-4 evaluation	2.80(0.63)	7.80(1.62)	17.10(1.91)	22.60(5.36)	48.70(2.54)	3.08(0.03)

Furthermore, we randomly sampled 100 samples from the MIMIC-CXR dataset and compared the score of the 5-point Likert scale between the GPT-4 and human evaluations, as shown in Table 6. An interesting observation from the study is that human annotators tend to have more neutral opinions; approximately 75% of their responses fall within the 2-3 range on a 5-point scale. However, GPT-4 frequently assigned a higher similarity score of 4 in 48.7% of cases. Using GPT-4 evaluation results in a significantly higher WASM score of 3.08, with a standard deviation of 0.03 across 10 runs, compared to a score of 2.24 when evaluated by humans. This raises the question of whether using GPT-4 for similar measurements provides a more robust metric for MRG tasks.

### Ablation study

We conducted an ablation study to explore the impacts of the two stage fine-tuning strategy on the VQA performance. In Tables 7 and  8, they show the VQA accuracy measurements of models that (i) perform zero-shot without fine-tuning; (ii) are only fine-tuned on the image caption dataset; (iii) are only fine-tuned on the VQA dataset; and (iv) have two stage fine-tuning.

**Table 7:** The ablation study under different training setups and the comparison between exact match evaluation, human evaluation and GPT-4 evaluation on the VQA-RAD dataset.

Evaluation methods	Stage 1	Stage 2	Open	Closed	Overall
(a) Exact match evaluation	$\times$	$\times$	13.4%	48.2%	34.4%
	$\checkmark$	$\times$	16.2%	59.9%	42.6%
	$\times$	$\checkmark$	58.1%	82.0%	72.5%
	$\checkmark$	$\checkmark$	**62.6%**	**87.1%**	**77.4%**
(b) GPT-4 evaluation	$\times$	$\times$	26.2%(1.1%)	49.1%(0.3%)	40.0%(0.4%)
	$\checkmark$	$\times$	37.3%(1.1%)	60.2%(0.1%)	51.1%(0.5%)
	$\times$	$\checkmark$	75.0%(0.4%)	82.7%(0.0%)	79.6%(0.1%)
	$\checkmark$	$\checkmark$	**79.9%(1.2%)**	**87.5%**(0.0%)	**84.4%(0.5%)**
(c) Human evaluation	$\checkmark$	$\checkmark$	**77.7%**(3.2%)	**87.6%(0.2%)**	**83.7%**(1.3%)

Significant values are in bold.

Tables 7 and  8 demonstrate that two-stage fine-tuning significantly enhances model performance across all three benchmark datasets in both exact-match and GPT-4 similarity evaluations, and second-stage fine-tuning contributes the most performance improvements. We primarily focus on the results from GPT-4 similarity evaluations, which are largely consistent with those from exact-match evaluations. Without fine-tuning the VQA-RAD dataset, models only achieve a 40% overall accuracy and 26.2% on open-ended questions. Stage 1 fine-tuning with image captioning tasks raises overall accuracy by 11.1%, while Stage 2 fine-tuning on VQA tasks alone doubles performance to 79.6% and boosts combined overall accuracy to 84.4%. Similarly, without fine-tuning, the Slake dataset starts with open-ended question accuracy at 47.0% and overall accuracy at 52.1%, with modest gains from image-caption task fine-tuning. Direct VQA task fine-tuning substantially improves Slake’s and PathVQA’s accuracies to 83.8% and 64.3%, respectively, with overall accuracies reaching 85.3% for Slake and 68.6% for PathVQA after completing both stages, notably increasing PathVQA’s open-ended question accuracy by 7.2%.

**Table 8:** The ablation study under different training setups and the comparison between exact match evaluation and GPT-4 evaluation on the Slake and PathVQA datasets.

Evaluation methods	Stage1	Stage2	Slake			PathVQA		
			Open	Closed	Overall	Open	Closed	Overall
(a) Exact match evaluation	$\times$	$\times$	23.9%	58.9%	37.6%	2.4%	59.0%	30.8%
	$\checkmark$	$\times$	11.6%	50.5%	26.9%	1.2%	53.3%	27.3%
	$\times$	$\checkmark$	77.5%	85.3%	80.6%	31.2%	90.0%	60.7%
	$\checkmark$	$\checkmark$	**77.8%**	**88.7%**	**82.1%**	**35.7%**	**91.3%**	**63.6%**
(b) GPT-4 evaluation	$\times$	$\times$	47.0%(0.5%)	59.9%(0.0%)	52.1%(0.3%)	12.3%	59.3%	35.9%
	$\checkmark$	$\times$	50.0%(0.9%)	62.5%(0.4%)	54.9%(0.7%)	12.1%	53.9%	33.1%
	$\times$	$\checkmark$	82.8%(0.3%)	85.3%(0.0%)	83.8%(0.2%)	38.5%	90.0%	64.3%
	$\checkmark$	$\checkmark$	**83.1%**(0.3%)	**88.7%**(0.0%)	**85.3%**(0.2%)	**45.7%**	**91.3%**	**68.6%**

*Negative Transfer and Task Interference:* We observe negative transfer effects when applying Stage-1 caption fine-tuning alone, which may introduce task interference for downstream Med-VQA. As shown in Table 8, fine-tuning only on image-caption data results in a performance drop on both SLAKE and PathVQA under exact-match evaluation. This degradation is particularly evident for open-ended questions, where the model is required to produce short and precise answers that align with the annotated ground truth.

We hypothesize that such negative transfer mainly arises from an objective mismatch between captioning and VQA: the captioning objective encourages descriptive and comprehensive generation, whereas Med-VQA often favors concise outputs (e.g. single-word or short-phrase answers). In addition, the caption datasets used in Stage 1 cover diverse medical image types and writing styles, which may bias the decoder toward narrative-like responses and reduce its sensitivity to question-specific constraints. This effect is amplified under lexical similarity metrics (exact match), where semantically correct but paraphrased answers may still be counted as incorrect.

Importantly, our two-stage adaptation pipeline alleviates this negative transfer by introducing task-specific supervision in Stage 2. After fine-tuning on the target VQA datasets, the model recovers from the caption-induced bias and achieves the best overall performance across all benchmarks (Tables 7 and 8). These results suggest that caption fine-tuning is beneficial as an intermediate adaptation step only when combined with downstream task training, rather than being used as a standalone objective.

### Clinical implications and transferability

The proposed PeFoMed framework provides a practical pathway for adapting multimodal large language models (MLLMs) to medical CXR applications under limited computational budgets. Clinically, such a parameter-efficient adaptation pipeline can support radiology workflows in multiple ways, including (i) assisting clinicians in image understanding through visual question answering, and (ii) generating draft radiology reports that can be reviewed and edited by radiologists to improve documentation efficiency. Importantly, we emphasize that the generated outputs are intended for clinical decision support rather than autonomous diagnosis, and should always be verified by qualified medical professionals.

Beyond the evaluated Med-VQA and MRG tasks, our approach is transferable to other medical vision-language tasks by modifying the task identifier tokens and instruction templates while keeping the majority of the backbone frozen. Specifically, PeFoMed freezes both the vision encoder and the LLM, and only updates the projection layer and LoRA parameters, which enables efficient task adaptation with a small trainable footprint. For example, the same architecture can be extended to: (1) multi-label abnormality classification by constraining the answer format to predefined clinical labels, (2) radiology report summarization or impression generation by using structured prompts (e.g. “Generate the impression section”), (3) image-text retrieval or clinical grounding by training with contrastive or ranking objectives.

In terms of modality generalization, while this study focuses on chest X-ray report generation, the two-stage fine-tuning strategy can be applied to other modalities such as CT, MRI, ultrasound, and pathology images by introducing modality-specific image-caption data in Stage 1 and task-specific supervision in Stage 2. This design allows the model to acquire domain terminology and imaging-specific descriptions before being specialized to downstream objectives. Future work will further validate the transferability of PeFoMed on additional clinical tasks and imaging modalities, and evaluate its reliability under real-world clinical distributions.

## Conclusion

In this work, we proposed a parameter-efficient fine-tuning framework for adapting multimodal large language models to Med-VQA and MRG tasks in a generative manner. We further observed that conventional lexical-similarity-based metrics may be insufficient for evaluating generated medical content. Therefore, we investigated GPT-4-based semantic evaluation as a supplementary assessment method and compared it with human evaluation and conventional metrics on both Med-VQA and MRG tasks. The findings indicate that the GPT-4 evaluation provides a useful supplementary semantic signal, especially for scalable analysis of generated outputs, but it should not replace human or expert evaluation, particularly for MRG.

## Limitation

Our research facilitated visual question answering and medical report generation in the chest X-ray domain. However, the model underperformed compared to specialized non-LLM models on the medical report generation (MRG) task. This discrepancy arises from the fact that the MRG task not only evaluates the accuracy of reports but also considers the length and structure of commonly occurring sequences, an aspect for which the model has not been adequately trained. Future work will explore more diverse medical report data and structure-aware training objectives, such as report-structure-aware or clinically guided loss functions, to better model the content organization and diagnostic emphasis of radiology reports.

## Supplementary Information

Supplementary Information 1.

Supplementary Information 2.

## Data Availability

The code and datasets generated during the current study are available here: https://github.com/jinlHe/PeFoMed
