# Supplementary material for: PeFoMed: Parameter efficient fine-tuning of multimodal large language models for medical CXR
Source: Sci Rep. 2026 Apr 21;16:18729. doi: 10.1038/s41598-026-47871-2 (PMC13273104; doi:10.1038/s41598-026-47871-2)
Supplement: Supplementary file 1 — Supplementary Information 1. [file 41598_2026_47871_MOESM1_ESM.pdf]

Appendix

A. Human Evaluation Protocol

Inter-rater reliability was assessed using Krippendorff's α with 95% bootstrap confidence intervals(B=1000 resamples, sampling items with replacement). We used α (ordinal) for report ratings since the 5-point Likert scale (0–4) is ordinal, and α (nominal) for VQA since answer correctness was judged in a binary manner (correct/incorrect). As expected, agreement is higher for the VQA setting due to its more objective decision boundary, whereas report-level semantic consistency is inherently more subjective.

Appenx Table 1. Inter-rater reliability (Krippendorff's α)

Task	Scale	α type	#Raters	α	95% CI
VQA	binary 0/1	nominal	10	0.807	[0.753, 0.842]
MRG	Likert 0–4	ordinal	10	0.698	[0.629, 0.756]

B. More Qualitative Results

We conducted detailed experiments on the Slake dataset, as illustrated in Figure 5. Experiments were carried out on various question types within the dataset. Notably, the "KG" type questions, those requiring additional knowledge for answers, exhibited the largest discrepancy post-evaluations. Possessing substantial additional knowledge precisely characterizes medical MLLMs. As indicated in Figure 6, experiments across various image organ types revealed that "Chest mediastinal" was the sole organ type yielding consistent results. This organ type is notably prevalent in the MIMIC-CXR dataset, which possesses the most extensive data used for first-stage image-caption fine-tuning. This observation further demonstrates the significant impact of stage 1 fine-tuning on downstream tasks.

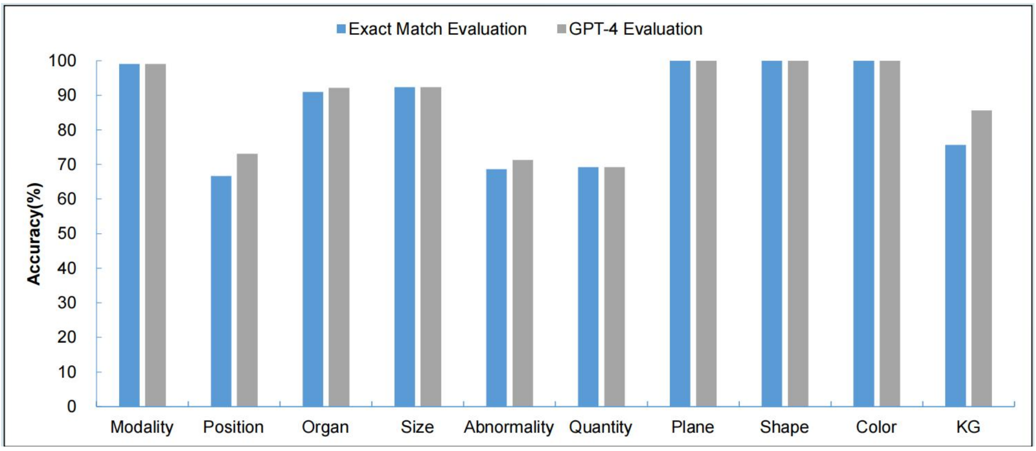

Figure5 The accuracy of different evaluation methods for different question types on the Slake dataset.

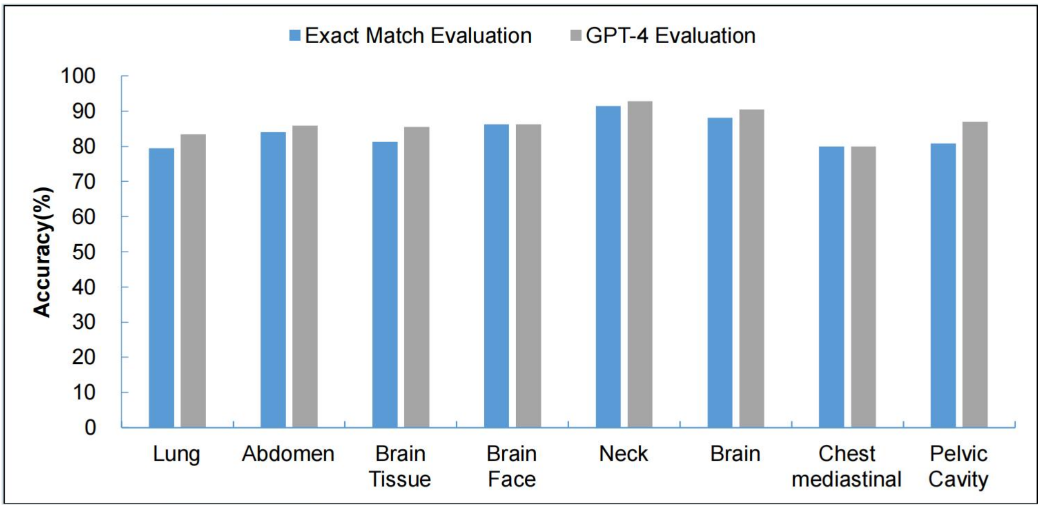

Figure6 The accuracy of different evaluation methods for different image organ types on the Slake dataset.

C. LLM-as-a-Judge Safeguards

As shown in Figures 7 and 8, we formulate instructions for GPT-4 evaluation using structured prompts and strict rule constraints to reduce potential biases and hallucinations introduced by the model. Here, Description i corresponds to the description for score i, Applicability i indicates when score i should be assigned, the actual medical report refers to the ground-truth report in the dataset, and the generated report refers to the report produced by our model.

Given an actual medical report on a medical image and a generated medical report that needs to be determined, evaluate the generated report to be determined (0 or 1 or 2 or 3 or 4).

standard :
4 : {Description 4}
Applicability:{Applicability 4}
3 : {Description 3}
Applicability:{Applicability 3}
2 : {Description 2}
Applicability:{Applicability 2}
1 : {Description 1}
Applicability:{Applicability 1}
0 : {Description 0}
Applicability:{Applicability 0}

actual medical report:
- actual medical report: {actual medical report}
generated medical report:
- generated medical report to be determined: {generated report}
Task:
- Given an actual medical report on a medical image and a generated medical report that needs to be determined, evaluate the generated report to be determined (0 or 1 or 2 or 3 or 4).

Hallucination Detection:
Verify that the generated medical report does not include hallucinated (inconsistent with input or facts) or unsupported medical claims. If the report makes unverifiable claims, output 0 for hallucination.

Output Format:
Score: your answer (0 to 4)
Hallucination: your answer (1 or 0)

Figure7 Instruction designed for GPT-4 to evaluate MRG.

Given a question about a medical image, there is a correct answer to the question and an answer to be determined. If the answer to be determined matches the correct answer or is a good enough answer to the question, output 1; otherwise output 0. Evaluate the answer to be determined (1 or 0).

Question:
- question about the medical image: {question}
Answers:
- correct answer (ground truth): {true answer}
- answer to be determined: {generated answer}

Task:
- Given a question about a medical image, there is a correct answer to the question and an answer to be determined. If the answer to be determined matches the correct answer or is a good enough answer to the question, output 1; otherwise output 0. Evaluate the answer to be determined (1 or 0).

Hallucination Detection:
Verify that the generated answer does not include hallucinated (inconsistent with input or facts) or unsupported medical claims. If the answer contains unverifiable claims, output 0 for hallucination.

Output Format:
Correctness: your answer
Hallucination: your answer (1 or 0)

Figure8 Instruction designed for GPT-4 to evaluate Med-VQA.
